# Misinterpretations about CT numbers, material decomposition, and elemental quantification

**DOI:** 10.1007/s00330-024-10934-x

**Published:** 2024-07-21

**Authors:** Aria M. Salyapongse, Timothy P. Szczykutowicz

**Affiliations:** 1 Department of Medical Physics, University of Wisconsin Madison, Madison, WI USA; 2 Department of Radiology, University of Wisconsin Madison, Madison, WI USA; 3 Department of Biomedical Engineering, University of Wisconsin Madison, Madison, WI USA

**Keywords:** Quantitative computed tomography, Bone mineral density, Iodinated contrast, Calcium quantification, Quantification error

## Abstract

**Background:**

Quantitative CT imaging, particularly iodine and calcium quantification, is an important CT-based biomarker.

**Purpose:**

This study quantifies sources of errors in quantitative CT imaging in both single-energy and spectral CT.

**Materials and methods:**

This work examines the theoretical relationship between CT numbers, linear attenuation coefficient, and material quantification. We derive four understandings: (1) CT numbers are not proportional with element mass in vivo, (2) CT numbers are proportional with element mass only when contained in a voxel of pure water, (3) iodine-water material decomposition is never accurate in vivo, and (4) for error-free material decomposition a voxel must only consist of the basis decomposition vectors. Misinterpretation-based errors are calculated using the National Institute of Standards and Technology (NIST) XCOM database for: tissue chemical compositions, clinical concentrations of hydroxyapatite (HAP), and iodine. Quantification errors are also demonstrated experimentally using phantoms.

**Results:**

In single-energy CT, misinterpretation-induced errors for HAP density in adipose, muscle, lung, soft tissue, and blood ranged from 0–132%, i.e., a mass error of 0–749 mg/cm^3^. In spectral CT, errors with iodine in the same tissues resulted in a range of < 0.1–33% error, resulting in a mass error of < 0.1–1.2 mg/mL.

**Conclusion:**

Our work demonstrates material quantification is fundamentally limited when measured in vivo due to measurement conditions differing from assumed and the errors are at or above detection limits for bone mineral density (BMD) and spectral iodine quantification. To define CT-derived biomarkers, the errors we demonstrate should either be avoided or built into uncertainty bounds.

**Clinical relevance statement:**

Improving error bounds in quantitative CT biomarkers, specifically in iodine and BMD quantification, could lead to improvements in clinical care aspects based on quantitative CT.

**Key Points:**

- *CT numbers are only proportional with element mass only when contained in a voxel of pure water, therefore iodine-water material decomposition is never accurate in vivo.*
- *Misinterpretation-induced errors ranged from 0–132% for HAP density and <* *0.1–33% in spectral CT with iodine*.
- *For error-free material decomposition, a voxel must only consist of the basis decomposition vectors*.

## Introduction

CT number is commonly used for diagnostics, disease progression tracking, and characterization [1]. It is also used as a method with which to determine the amount of an element or molecule of interest in a tissue or phantom [2]. Specifically, CT number is used for calculating the calcium score in coronary artery scans [3, 4], the bone mineral density (BMD) in osteoporosis, rheumatoid arthritis, Crohn’s disease, and myeloma studies [5, 6], and iodine uptake [7, 8].

Material or elemental quantification (e.g., hydroxyapatite (HAP) or iodine) methods rely on assumptions about CT numbers, linear attenuation coefficients, and element density. Namely, that the relationships between these variables are proportional in vivo. If fundamental misinterpretations of the relationships between an element’s mass and CT number exist, these misinterpretations could potentially explain iodine and HAP quantification errors. For example, Brunnquell et al 2021, in a BMD literature review analysis found HAP quantification errors remaining of 4–6% even when a synchronous calibration procedure was employed [9]. Understanding the limits of these assumptions is needed for CT to become a biomarker creator; currently no quantitative imaging and biomarker alliance (QIBA) profile for CT number accuracy or material density exists [10].

In this work, we endeavor to dismantle this assumption of proportionality and show that its use in clinical and phantom studies may lead to nonnegligible errors on elemental (e.g., calcium or iodine) mass. In doing so, we illustrate four key understandings: (1) CT numbers are not proportional with element mass in vivo, (2) CT numbers are proportional with element mass only when contained in a voxel of pure water, (3) iodine-water material decomposition is never accurate in vivo, and (4) for error-free material decomposition a voxel must only consist of the basis decomposition vectors. We also quantify the errors due to misunderstanding the above key understandings.

## Materials and methods

To illustrate the errors that are inherent in both single-energy and spectral material decomposition in CT, let us consider a voxel composed of some material (e.g., soft tissue, bone, adipose tissue) and a material of interest (MOI, e.g., iodine or HAP) and the mass of the MOI is calculated. As is shown below, the mass of the MOI that is calculated from CT number (single-energy CT) or material density maps (spectral CT) has inherent errors due to assumptions on the background material. In the following work, it will be shown that the underlying assumption in CT material quantification, that the background material is water, is the cause of some of the errors in CT material quantification, and that this is never true in vivo.

### Single-energy error derivation

First, to demonstrate how single-energy CT material quantification is prone to error, it is necessary to begin with some equations that describe CT material quantification. Equations 1–3 are these equations.

1 $$C{T}_{{{\#}}}=\frac{\mu -{\mu }_{{water}}}{{\mu }_{{water}}}\cdot 1000$$

2 $$V{F}_{B}=1-V{F}_{{MOI}}$$

3 $${m}_{{element}}=V{F}_{{MOI}}\cdot {\rho }_{{MOI}}\cdot M{F}_{{element}}\cdot {VV}$$

Equation 1 is the classic definition of CT number [11] where *CT*_*#*_ is the CT number in Hounsfield units, $\mu$ is the linear attenuation coefficient of a voxel, and ${\mu }_{{water}}$ is the linear attenuation coefficient of water in units of cm^−1^. Equation 2 describes the conservation of volume for a voxel made of some background material (B) and MOI like HAP or iodine contrast material (ICM). VF_B_ is the volume fraction of the background material, while VF_MOI_ is the volume fraction of the MOI. Usually, elements the radiology community cares about are not found in the body in pure element form. For example, iodine is delivered bound inside a molecule and mixed with other stabilizing agents. Therefore, we include Eq. 3 to describe the mass of an element inside a MOI. Equation 3 describes the mass of an element (*m*_*element*_) in a voxel with a given VF_MOI_, MOI density (*ρ*_*MOI*_), mass fraction of the element within the MOI (*MF*_*element*_), and the volume of the voxel (*VV*).

To examine the error in single-energy CT material quantification, a voxel of some background material and an MOI is constructed.

4 $$\mu =V{F}_{{MOI}}\cdot {\mu }_{{MOI}}+V{F}_{B}\cdot {\mu }_{B}$$

For example, a voxel with VF_MOI_ = 0.01 and VF_B_ = 0.99 means that 1% of the volume of the voxel is made of the MOI and 99% of the voxel is made of the background material. For a voxel with an MOI of 350 mgI/cm^3^ (we used the specific chemical formulation of OMNIPAQUE^TM^ 350, GE Healthcare, London UK) [12], VF_MOI_ between 0 and 0.10 represents an iodine density between 0 g/cm^3^ and 0.035 g/cm^3^, which is the range of clinical iodine densities present in the body during contrast-enhanced scans [13, 14].

By inserting Eqs. 3 and 4 into Eq. 1 using the definition of volume conservation (Eq. 2), we derive a general expression for CT numbers for the condition of a voxel containing MOI and background material as,

5 $$C{T}_{{{\#}}}=\frac{{m}_{{element}}}{{\rho }_{{MOI}}\cdot M{F}_{{element}}\cdot {VV}}\cdot \frac{{\mu }_{{MOI}}-{\mu }_{B}}{{\mu }_{{water}}}\cdot 1000+\frac{{\mu }_{B}-{\mu }_{{water}}}{{\mu }_{{water}}}\cdot 1000$$

From this, it can be seen that there are two terms that make up the CT number in Eq. 5. The second term is the standard CT number equation for a voxel containing only the background material, but the first term is linear with the mass of the element in the voxel. It becomes apparent this expression demonstrates CT numbers are not proportional to element mass unless the background material is pure water. Equation 5 demonstrates CT numbers are linear with element mass. While both a proportional and linear relationship reflects a “straight line” relationship between two quantities, only a proportional relationship allows a percent change in one quantity to be mirrored in the other quantity.

In the case where the background material is water, the second term of Eq. 5 drops out, and only the concentration of the element determines the CT number. This demonstrates that the widely held belief that CT numbers are proportional to element mass holds true only when the background material of the voxel containing the element is water.

### Single-energy error quantification

The error in elemental mass can be quantified as,

6 $${Percent\; elemental\; error}=\frac{{m}_{{assumed}}-{m}_{{real}}}{{m}_{{real}}}\cdot 100 \%$$

Where the assumption for the mass (*m*_*assumed*_) uses the standard proportional relationship historically applied in the radiology community [4, 15] where CT_#1_ is the measured CT number of a voxel containing the original mass (m_1_) and CT_#2_ corresponds to a voxel with the new mass (m_2_),

7 $${m}_{2}=\frac{C{T}_{{{\#}}2}}{{{CT}}_{{{\#}}1}}{m}_{1}.$$

Equation 7 assumes incorrectly that ${m}_{2}$ is equal to the ratio of CT numbers multiplied by ${m}_{1}$. To evaluate the difference between Eq. 7 and Eq. 5, it can be assumed that there is a starting mass $\left({m}_{1}\right)$ in the voxel which increases by a factor $X$ as,

8 $${m}_{2}=X\cdot {m}_{1}$$

**Fig. 1:**
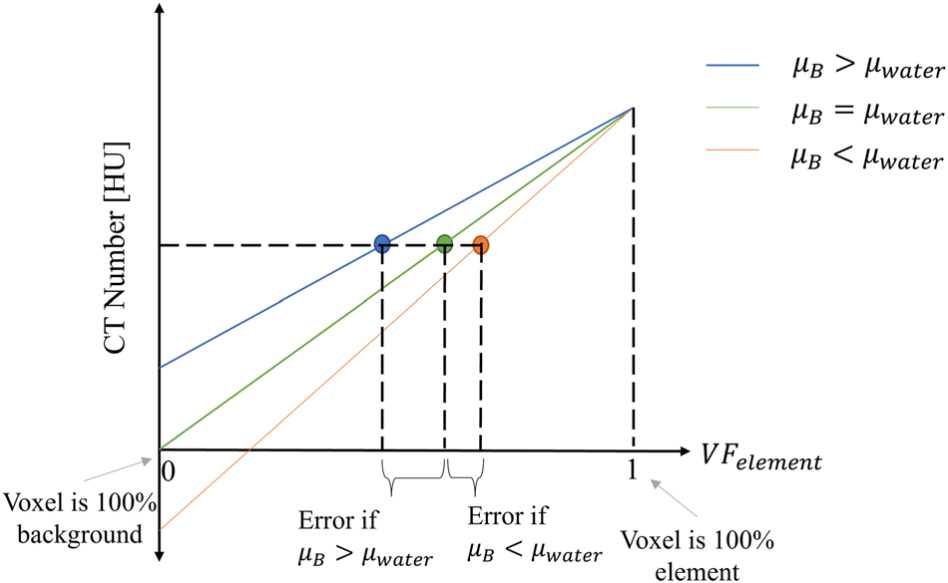
A graphical representation of the error on the volume fraction (i.e., the element’s mass) of the element under consideration. If ${\mu }_{B}$ (the linear attenuation coefficient of the background material within the voxel) is greater than ${\mu }_{{water}}$ (the linear attenuation coefficient of water in the voxel) then the error will be positive (i.e., the actual element mass is less than the assumed element mass). This relationship reverses if ${\mu }_{B} \, < \, {\mu }_{{water}}$. The only way there is no error on the element mass is if the background material in the voxel is water

The assumed iodine mass requires the calculation of the original and final CT numbers. See the Supplementary Materials for a derivation of error as a function of X. The error can be understood graphically with Fig. 1.

To demonstrate these errors, we performed a simulation with several different background materials and clinical HAP masses [4]. This simulation used tissue compositions and standard densities from reference [16] to compose a voxel (1 cm^3^) of known tissue (i.e., background) and HAP volumes such that the mass of HAP in the voxel followed Eq. 3. We varied the change in HAP mass (*X* in Eq. 8) from 0.5 to 3.5 in steps of 0.5 with background voxel materials including vacuum, adipose tissue, water, muscle, lung, soft tissue, blood, and cortical bone (bone) with an initial HAP mass of 0.126 g (m_1_ = 0.126 g HAP). Mass attenuation coefficients were calculated using the National Institute of Standards and Technology’s (NIST) XCOM [17] for the different voxel compositions at 70 keV. Neither noise model, image reconstruction procedure, nor beam hardening correction was applied.

We also scanned a phantom commonly used for HAP quantification (Cardio Calcium Scoring Phantom, QRM GmbH, Moehrendorf, Germany) [4] on a clinical CT system (Discovery HD 750, GE Healthcare Waukesha, WI) and measured the mean CT number for the different HAP density inserts. The QRM phantom comprises an anthropomorphic thorax section measuring 30 cm across, 20 cm tall, and 10 cm deep made of tissue and lung equivalent material. The central region also contains nine HAP inserts of three varying concentrations (200, 400, 800 mg/cm^3^) and sizes. We acquired data at 120 kV, 500 mA, reconstructed images at 0.625 mm slice thickness, a “Detail” reconstruction filter, a pitch of 0.984:1, rotation time of 0.4 s, and a 40 mm beam collimation, which gives a CTDI_vol_ of 16.3 mGy. Eight slices were averaged for each density insert using region of interest (ROIs) of 19.6 mm^2^ (50 pixels).

### Spectral CT error derivation

Spectral scanning takes advantage of the fact that linear attenuation coefficients can be described as a linear combination of the linear attenuation coefficients due to the photoelectric effect and the Compton scatter [18] or material basis vectors [19].

The primary and well-known misinterpretation that occurs when viewing basis map images is that the information given on a particular voxel is made of the basis vectors from which it is composed. For example, a voxel of calcium can be decomposed into the water and iodine basis, but the resulting material density maps cannot be interpreted as depicting the voxel’s true water or iodine composition [20, 21].

**Fig. 2:**
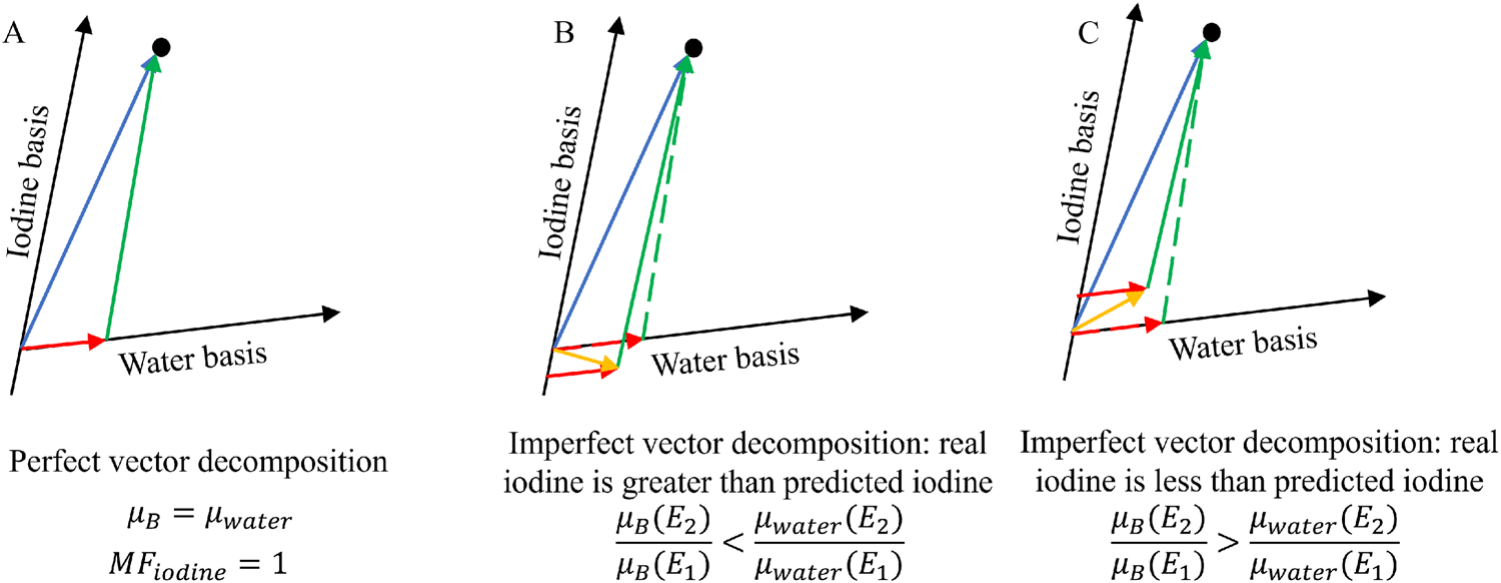
Graphical representation of the three cases for dual-energy decomposition of a voxel in an iodine-water basis. Case A exhibits no iodine quantification error but doesn’t represent clinical reality. In **B** and **C**, the material decomposition provides an erroneous estimate of the true iodine density. **A** The voxel is made of elemental iodine and water; this results in perfect decomposition and no associated error in the iodine mass. **B** The voxel is made of a non-water background with a dual-energy ratio less than water ($\frac{{\mu }_{B}({E}_{2})}{{\mu }_{B}({E}_{1})} < \frac{{\mu }_{{water}}({E}_{2})}{{\mu }_{{water}}({E}_{1})}$) and ICM. **C** The voxel is made of a non-water background with a dual-energy ratio greater than water ($\frac{{\mu }_{B}({E}_{2})}{{\mu }_{B}({E}_{1})} > \frac{{\mu }_{{water}}({E}_{2})}{{\mu }_{{water}}({E}_{1})}$) and ICM. Blue line: vector of the voxel in the iodine-water basis. Green lines: assumed iodine (dashed), actual iodine (solid). Red lines: assumed water (dashed), actual water (solid). Yellow line: actual non-water background

In the sections that follow, we add an understanding to the known misinterpretation present in spectral decomposition. See Fig. 2 for a graphical explanation. Iodine-water material decomposition is never accurate in vivo, and error-free material decomposition in a voxel requires that both basis decomposition vectors exactly match the content of the voxel. Unlike the case of a voxel with no iodine present that appears in both the iodine-water and water-iodine image, this case can be trickier for interpretation because iodine is present, but the iodine vector has a contribution from non-iodine sources. This misinterpretation occurs in all material basis pairs (e.g., iodine-water, calcium-water, etc.).

Material or elemental quantification in spectral CT involves multiplying the mass density of a basis by the vector decomposition value for that basis. For example, we can define ${\mu }_{{voxel}}\left({E}_{i}\right)$, the attenuation of a voxel, as,

9 $${\mu }_{{voxel}}\left({E}_{i}\right)=a\cdot {\mu }_{{basis}1}\left({E}_{i}\right)+b\cdot {\mu }_{{basis}2}\left({E}_{i}\right),$$

where ${\mu }_{{basis}1}$ and ${\mu }_{{basis}2}$ are the basis vectors. Contrary to the common interpretation, the exact value of “$a$” is dependent on more than just the amount of basis 1 in the voxel. The “$a$” term is dependent on the background material, the amount of element in the MOI, and the basis 1 and 2 choices as shown graphically in Fig. 2. See the Supplementary Materials for a detailed examination of exactly how the “$a$” term is dependent on these factors.

### Spectral CT error quantification

In the spectral decomposition case, taking the “$a$” term from Eq. 9 and the basis 1-basis 2 image and multiplying “$a$” by elemental (basis 1) density and voxel volume to get mass implicitly assumes two things: (1) that the background material is basis 2, and (2) that the MOI is actually elemental basis 1. These assumptions are always violated in vivo. The explicit form of the error quantification equation is derived in the Supplementary Materials.

To demonstrate these errors, we performed a simulation with several different background materials and clinical iodine volume fractions [13, 14]. We varied the volume fraction ($V{F}_{{ICM}}$) of the ICM from 0.01 to 0.10 (0.01, 0.02, 0.05, 0.08, 0.10) with background voxel materials including vacuum, adipose tissue, water, muscle, lung, soft tissue, blood, and bone. Tissue compositions and standard densities are taken from reference [16] and mass attenuation coefficients are calculated using NIST XCOM [17]. Energy level 1 was assumed to be 58 keV, and energy level 2 was assumed to be 85 keV (which are the estimated average beam energies for 80 and 140 kV, respectively [22, 23]).

We also scanned a phantom commonly used for iodine quantification (Multi-Energy CT Phantom, Sun Nuclear) on a clinical CT system (Discovery HD 750, GE Healthcare Waukesha, WI) and measured iodine density on material decomposition maps. We acquired data at 80 kV/140 kV with fast kV switching, reconstructed images at 5 mm slice thickness, a “Detail” reconstruction filter, a pitch of 0.531:1, rotation time of 1 s, and a 40 mm beam collimation, which gave a CTDI_vol_ of 58.14 mGy. Five slices were averaged for each density insert using ROIs of 20 mm^2^ (30 pixels).

## Results

### Error quantification with different tissues

**Fig. 3:**
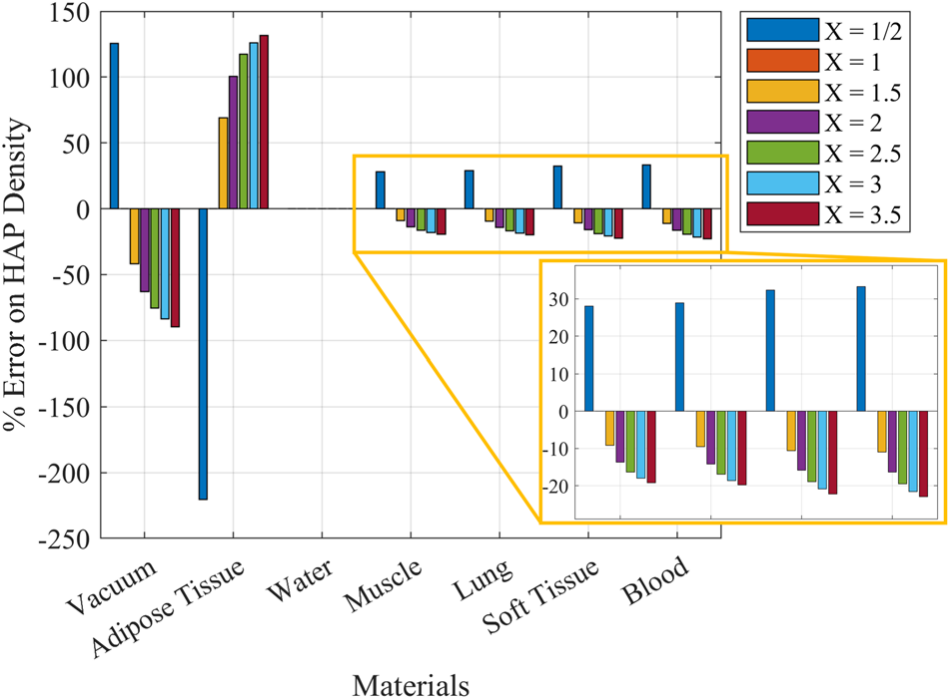
The error on the mass calculation of hydroxyapatite (HAP) when the mass has been increased by different X factors (e.g., X = 2 means the HAP mass has doubled)

To examine the single-energy CT elemental error, we plot (Fig. 3) the error using Equation A3 with different values of X, different background materials, and an MOI of HAP. When X < 1, the error is positive, and when X > 1, the error is negative (except for adipose tissue, where the relationship is reversed). X = 1 has no error (as expected) and water also never has an error (also expected). For materials far from water (like vacuum and adipose tissue) the error can approach or overtake 100%.

**Fig. 4:**
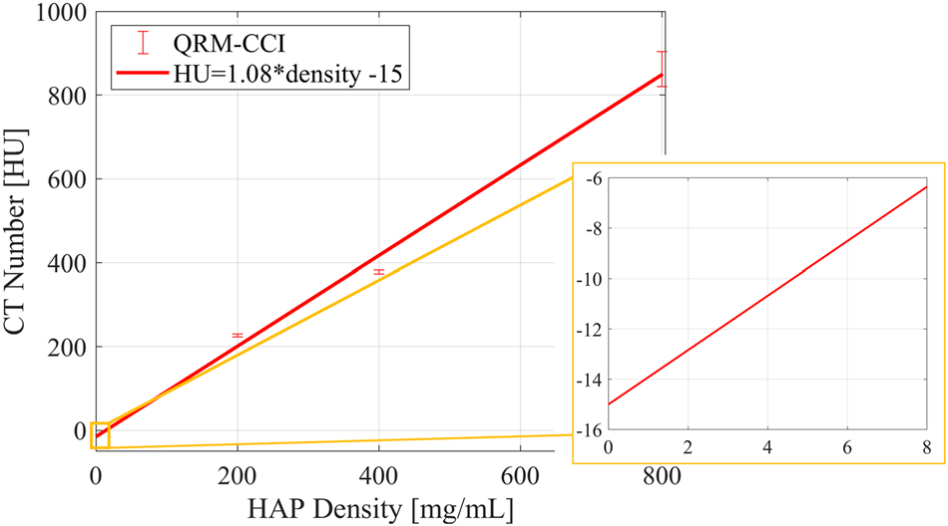
Plot of HAP CT number versus HAP density for a phantom commonly used for Ca quantification

Figure 4 plots the mean CT number of the three largest HAP inserts in the QRM phantom. This figure demonstrates how even phantoms used in quantitative CT do not exhibit a proportional relationship between CT number and material density. The inset graph and the linear regression fit parameters make it clear that the y-intercept is non-zero, meaning that the relationship between HAP density and CT numbers is linear but not proportional as demonstrated by Eq. 5. Use of the phantom will lead to erroneous HAP quantification because (1) HAP doesn’t occur mixed with water in vivo and (2) while the QRM phantom background more closely mimics in vivo coronary plaque HAP backgrounds, CT numbers are normalized to water, not fat.

**Fig. 5:**
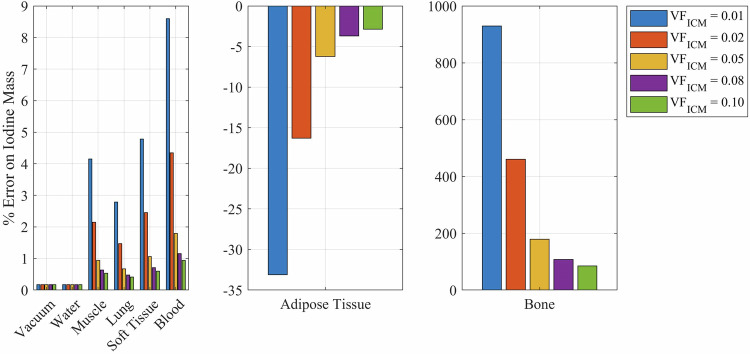
The error on the iodine mass in a voxel when the assumptions of (1) the voxel containing only iodine and water, and (2) the ICM being pure elemental iodine are violated

To examine spectral CT elemental mass error, we plot (Fig. 5) the error using Equation A5 with different values of “$a$” (i.e., different $V{F}_{{ICM}}$ values) and different background materials. The difference here from the single-energy case (Fig. 3) as even with a background material of water, there is still an error in the mass of iodine due to the ICM not being pure iodine. These errors are smaller for most background materials (vacuum, water, muscle, lung, soft tissue, and blood) compared with single-energy CT elemental error. However, the error on iodine density in a voxel with a background of bone approaches 1000%.

**Fig. 6:**
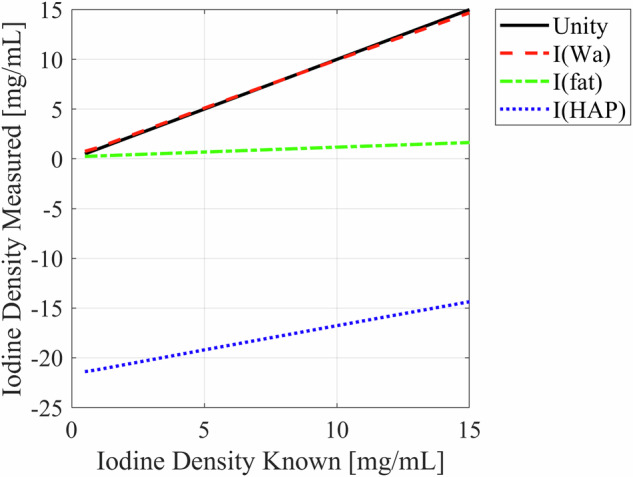
Measured iodine density versus known iodine density. The black line represents unity, which corresponds to perfect iodine measurement which is limited to the non-clinical condition of the background containing pure water. The red dashed line represents iodine-water subtraction (i.e., a water background). The red line is near unity because the MECT phantom uses a water background in its iodine inserts. The green dashed line represents iodine-fat subtraction. The blue dotted line represents iodine-HAP subtraction. This graph demonstrates that an incorrect background assumption can drastically change the assumed iodine quantity in the voxel

We also used a phantom to demonstrate this error. Figure 6 plots the iodine quantification (i.e., the “$a$” term) in material decomposition images of the MECT phantom (Multi-Energy CT Phantom, Sun Nuclear Corporation, Melbourne, FL). This figure demonstrates how a different assumption on the background material impacts the measured amount of iodine in a voxel.

## Discussion

In this work, we have detailed the historical precedence for misinterpretation surrounding CT numbers and element density proportionality in single-energy CT. In spectral CT, we have identified a misinterpretation; an accurate decomposition requires a voxel to contain only the basis materials. In other words, a voxel containing iodine will only provide an accurate iodine density when it resides in a pure water mixture (i.e., an inaccurate in vivo condition) or the background voxel composition is known and chosen as a decomposition basis partner with iodine. A possible source of these misinterpretations in our field is due to the prevalence of water-based phantom models. As we have shown, a water-based phantom would result in very small errors on the elemental mass. If water-based phantom results were then used to justify applying this quantification technique in clinical practice, this would lead to erroneous elemental quantifications.

It is important to note that the errors expressed in this work would still be present in 3-material decomposition because the fundamental misunderstanding(s) that lead to error is the mismatch between voxel composition and basis vectors. For example, if a voxel contains soft tissue, fat, and iodinated-contrast material and the three basis vectors chosen were soft tissue, fat, and elemental iodine, there would still be an error in the calculated iodine mass. In addition, photon-counting detectors would not mitigate this issue. Photon-counting detectors improve noise and spatial resolution and give inherent spectral information in the resulting scans. However, neither noise, spatial resolution, nor the presence of spectral information is the source of error described in this work. Therefore, the use of photon-counting detectors would not change the fundamental misunderstandings presented in this work. Finally, the errors in CT number and material/element quantification described in this paper are distinct from other known sources of CT number error. For example, CT numbers are well known to change with acquisition beam energy [23], CT vendor [24], position within the gantry [25], patient size [1], and beam hardening [26].

While our examples focus on iodine and calcium in a voxel, this work is applicable to any element or molecule in CT imaging.

It is common in the literature surrounding quantitative CT to use the ratio of CT numbers, which assumes that the relationship between CT numbers and elemental density is proportional. For example, in McCollough et al 2007, “[t]o obtain absolute values for calcium mass (m), a calibration measurement is necessary. The CT number of a calcification with known HA density (ρHA) is measured, and a calibration factor (c) is determined such that ${m}_{i}=c\cdot C{T}_{i}\cdot {V}_{i}$” [4]. Adding a calibration factor, (c), to the relationship between mass and CT numbers implies proportionality. Let us examine what happens to the mass if we assume an (incorrect) proportional relationship between CT numbers and elemental mass. Suppose we have a sample of soft tissue mixed with 0.126 g of HAP. We record $C{T}_{\#1}=165{HU}$, then increase to a HAP mass of 0.311 g yielding $C{T}_{\#2}=330{HU}.$ Using the conventional methodology of determining HAP concentration, we would we assume that the HAP mass change is equivalent to the CT number change. A CT number ratio of $\frac{330{HU}}{165{HU}}=2$, so we would incorrectly assume that we have a doubling of HAP mass in the voxel when in fact we increased HAP by 2.5 times. Details for this example are provided in the Supplementary Materials.

To mitigate error in clinical practice, it is important to base mass calculations on linear attenuation coefficients in the single-energy case. The spectral decomposition case is more complicated, as a user is limited to what the vendor picks to build the basis pairs from (e.g., iodine, water, calcium, HAP, etc.). Knowing a priori what the voxel is approximately made of before iodine is present could allow a user to tune the non-iodine basis pair to match the background voxel composition to mitigate error. Another method is to use multiple-phase imaging of a non-contrast and then a contrast-enhanced scan and use the non-contrast scan to calibrate the basis and remove the background material contribution.

Quantitative CT scanning is useful in assessing BMD, as well as screening for osteoporosis and estimating bone strength [5, 6, 9]. However, as noted by Brunnquell et al 2021, the measurement of BMD is not a trivial task and can be hampered by error sources including scanner-source errors (longitudinal instability, inter-scanner differences), technique-source errors (acquisition and reconstruction parameters, resolution), as well as patient-source errors (beam hardening, mispositioning, contrast agents, metal) [9]. The errors we demonstrate in HAP quantification of clinical tissues due to the misinterpretation presented in this work are on the order of 0–132%, which is a much larger range than that reported by Brunnquell et al 2021. Brunnquell et al 2021, in a BMD literature review analysis, found HAP quantification errors between 4% and 6% when using a synchronous calibration of 50–100 mg/cm^3^, while with higher density inserts of 200 mg/cm^3^ had lower errors around 3.7% [9]. Therefore, in assessing BMD, it is essential to be aware of the misinterpretation in order to make BMD assessments as accurate as possible.

Jacobsen et al (2019) reported on the lower limits of iodine detection and quantification in dual-energy (i.e., spectral) CT on several clinical systems [8]. Jacobsen et al (2019) reported that the limit of detection (LOD) was 0.021–0.257 mg of iodine per milliliter in their small phantom, and 0.026–0.547 in their larger phantom [8]. Our results indicate that errors in iodine quantification are on the order of a few percent for most clinically relevant tissues. Specifically, for clinically relevant concentrations of iodine (0–35 mg/mL), this means a range of < 1–9%, which is a range of 0.03–0.35 mg of iodine. These values are comparable the LOD reported by Jacobsen et al (2019), which indicates that the errors caused by incorrect assumptions detailed above are not negligible. McCollough et al 2007 [4] reviewed methods for HAP quantification. In the review, McCollough et al 2007 [4] describe a method for correcting a calibration phantom’s CT number to adjust for non-zero water background. This adjustment would correct for the misinterpretation described in this paper if two conditions were met: (1) HAP in vivo was present in a water background (which doesn’t reflect clinical reality) and (2) the water background correction was also applied to the measured CT number of HAP + water voxel measurements (in McCollough et al 2007 the correction is only applied to the calibration factor measurement [4]).

Some vendors appear to be aware of the errors associated with misunderstandings of the key points and have implemented some algorithms for specific or special indications to account for this. Specifically, Siemens Healthineers has a vendor-specific workflow for the characterization of urinary stones (Syngo CT Dual Energy/Kidney Stones, Siemens Healthineers) that utilizes a subtraction of a kidney background (i.e., they do not assume a pure water background) and compares the stones’ dual-energy ratio (the ratio of CT attenuation at low- and high-energy spectra) to characterize uric acid and non-uric acid stones [27]. Similarly, Siemens Healthineers also have a specific three-material decomposition (Syngo, Dual Energy Liver VNC, Siemens Healthineers) for virtual unenhanced images of the liver that assumes a fat/soft-tissue background (i.e., they do not assume a pure water background) [28]. These approaches both attempt to mitigate the errors associated with misunderstanding the key points presented in this paper and demonstrate concrete steps vendors and users can take to minimize errors.

The limitations of this work are primarily due to the narrow scope of this work. We only include material quantification errors due to background material violating the assumption that the background is made of water. Other errors on CT material quantification due to beam hardening, patient positioning, kV choice, CT vendor, and patient size are not included [1, 23–26, 29, 30]. All of these factors will make the errors presented here even worse. Specifically, the choice of CT vendors to implement different mathematics of beam hardening corrections and material quantification will affect these results [28]. In the practice of quantitative CT, there are more unknowns in clinical practice than we can quantify due to the vendors’ proprietary data manipulation steps.

This work is particularly important as the use of quantitative CT imaging increases. The Quantitative Imaging Biomarkers Alliance (QIBA) was formed by the Radiological Society of North America (RNSA) in 2007 in order to “improve the value and practicality of quantitative imaging biomarkers” [10]. The presence of this group indicates the utility of quantitative imaging, and our work indicates that the misinterpretation outlined in this work can impact this utility. By being aware of these misinterpretations, a radiologist or medical physicist can be more confident in understanding the measurement from quantitative CT imaging, which is in line with the mission statement of QIBA. To our knowledge, no current QIBA profile covers CT numbers in single or spectral imaging. Avoidance or awareness of the misinterpretations presented in this work should help QIBA efforts in CT number-based biomarker profiles.

## Supplementary information

ELECTRONIC SUPPLEMENTARY MATERIAL

## Abbreviations

BMD: Bone mineral density
HAP: Hydroxyapatite
ICM: Iodinated contrast media
MOI: Material of interest
NIST: National Institute of Standards and Technology
QIBA: Quantitative imaging biomarkers alliance
ROI: Region of interest
