# Supplementary material for: Misinterpretations about CT numbers, material decomposition, and elemental quantification
Source: Eur Radiol. 2024 Jul 21;35(2):862–70. doi: 10.1007/s00330-024-10934-x (PMC11782396; doi:10.1007/s00330-024-10934-x)
Supplement: Supplementary file 1 — ELECTRONIC SUPPLEMENTARY MATERIAL [file 330_2024_10934_MOESM1_ESM.pdf]

Misinterpretations about CT Numbers, Material Decomposition, and Elemental Quantification

ELECTRONIC SUPPLEMENTARY MATERIAL

Single energy error quantification

To examine elemental mass error as in Figure 3, we need to express Equation 6 in terms of known quantities: original CT number ($CT_{\#1}$), original mass (m_1), background material (μ_B), change in mass (X), and new CT number ($CT_{\#2}$). Inserting equations 7 and 8 into 5,

$$CT_{\#1} = \frac{m_1}{\rho_{MOI} \cdot MF_{element} \cdot VV} \cdot \frac{\mu_{MOI} - \mu_B}{\mu_{H_2O}} \cdot 1000 + \frac{\mu_B - \mu_{H_2O}}{\mu_{H_2O}} \cdot 1000, \quad (S1)$$

$$CT_{\#2} = \frac{X \cdot m_1}{\rho_{MOI} \cdot MF_{element} \cdot VV} \cdot \frac{\mu_{MOI} - \mu_B}{\mu_{H_2O}} \cdot 1000 + \frac{\mu_B - \mu_{H_2O}}{\mu_{H_2O}} \cdot 1000. \quad (S2)$$

Inserting Equations S1, and S2 in Equation 6, we get Equation S3. In Equation S3, we have kept $CT_{\#2}$ in the error equation because in practice one’s inputs to calculating an unknown voxel’s mass are calibration phantoms mass (m_1), the calibration phantoms CT number ($CT_{\#1}$), and the measured CT number ($CT_{\#2}$).

$$Elemental\ error = \frac{(1 - X)(\mu_B - \mu_{H_2O}) \cdot 1000}{CT_{\#2} \cdot \mu_{H_2O} - (1 - X)\left(\mu_B - \mu_{H_2O}\right) \cdot 1000} \cdot 100\% \quad (S3)$$

Different choices of X and background material are plotted in Figure 3.

Spectral CT error quantification

Let us examine a voxel of MOI (which contains the element under consideration) and background material content and examine its decomposition into water and element basis vectors. We begin with the definition of a voxel as in Equation 9, next, we write a system of equations relating the $\mu_{voxel}(E_i)$ to the bases and the MOI and background material basis at two energies (E_1 and E_2, corresponding to the effective low and high energies used in a dual energy decomposition) to solve for the coefficients “a” and “b”. We can use Equation 2 to describe the volume conservation within the voxel, and by adding energy dependence to Equation 4 as,

$$\mu_{voxel}(E_i) = VF_{MOI} \cdot \mu_{MOI}(E_i) + VF_B \cdot \mu_B(E_i). \quad (S4)$$

We can similarly add the energy dependence into Equation 9 as,

$$\mu_{voxel}(E_i) = a \cdot \mu_{basis\,1}(E_i) + b \cdot \mu_{basis\,2}(E_i). \quad (S5)$$

Next, we write a system of equations relating the $\mu_{voxel}(E_i)$ to the bases and the MOI and background material basis at two energies (E_1 and E_2) to solve for the coefficients “a” and “b”. Inserting Equation 2 into S4, and setting S4 equal to S5 for the low and high energies we obtain,

$$\begin{bmatrix} \varepsilon \\ \delta \end{bmatrix} = \begin{bmatrix} \mu_{basis\,1}(E_1) & \mu_{basis\,2}(E_1) \\ \mu_{basis\,1}(E_2) & \mu_{basis\,2}(E_2) \end{bmatrix} \begin{bmatrix} a \\ b \end{bmatrix}, \quad (S6)$$

Where $\varepsilon = (1 - VF_{MOI})\mu_B(E_1) + VF_{MOI} \cdot \mu_{MOI}(E_1)$ and $\delta = (1 - VF_{MOI})\mu_B(E_2) + VF_{MOI} \cdot \mu_{MOI}(E_2)$.

Solving this system of equations, we obtain:

$$a = \frac{\varepsilon \cdot \mu_{basis\,2}(E_2) - \delta \cdot \mu_{basis\,2}(E_1)}{\mu_{basis\,2}(E_2) \cdot \mu_{basis\,1}(E_1) - \mu_{basis\,2}(E_1) \cdot \mu_{basis\,1}(E_2)} \quad (S7)$$

$$b = \frac{\delta \cdot \mu_{basis\,1}(E_1) - \varepsilon \cdot \mu_{basis\,1}(E_2)}{\mu_{basis\,2}(E_2) \cdot \mu_{basis\,1}(E_1) - \mu_{basis\,2}(E_1) \cdot \mu_{basis\,1}(E_2)} \quad (S8)$$

In the case where the background material is water, the MOI is ICM, and the decomposition vectors are iodine and water, “a” and “b” reduce to:

$$a = VF_{ICM} \frac{\mu_{H_2O}(E_2) \cdot \mu_{ICM}(E_1) - \mu_{H_2O}(E_1) \cdot \mu_{ICM}(E_2)}{\mu_{H_2O}(E_2) \cdot \mu_{iodine}(E_1) - \mu_{H_2O}(E_1) \cdot \mu_{iodine}(E_2)} \quad (S9)$$

$$b = VF_{ICM} \left[\frac{-\mu_{iodine}(E_2) \cdot \mu_{ICM}(E_1) + \mu_{iodine}(E_1) \cdot \mu_{ICM}(E_2)}{\mu_{H_2O}(E_2) \cdot \mu_{iodine}(E_1) - \mu_{H_2O}(E_1) \cdot \mu_{iodine}(E_2)} - 1 \right] + 1 \quad (S10)$$

Equation S9 demonstrates that the “a” term is dependent on more than just the mass of iodine in the voxel. Equation S9 makes it clear that iodine mass calculations are a function of: actual iodine mass in the voxel, the non-iodine containing basis choice, and

the choice of iodine contrast agent and its relationship to elemental iodine concentration and basis 2.

In the limit where the background material is water and the MOI is actually elemental iodine, we can see that a and b further reduce to:

$$a = VF_{ICM} \quad (S11)$$

$$b = 1 - VF_{ICM} = VF_B \quad (S12)$$

Spectral CT error quantification

In current clinical practice, dual energy material decomposition-based iodine quantification assumes iodine mass density is determined via multiplication of the “a” term by a known calibration density of iodine as,

$$m_{assumed} = a \cdot \rho_{iodine} \cdot VV, \quad (S13)$$

Where VV is the voxel volume. Let us then examine the error on the mass calculation when Equation S14 is used. We calculate error using basis 1 of iodine, basis 2 of water, and an MOI of ICM (OMNIPAQUE^{TM} 350 [1]).

In can be shown that the real mass of iodine is given by

$$m_{real} = \frac{a-\tau}{\omega} \cdot \rho_{ICM} \cdot MF_{iodine} \cdot VV, \qquad (S14)$$

where,

$$\tau = \frac{\mu_{basis\,2}(E_2)\cdot\mu_B(E_1) - \mu_{basis\,2}(E_1)\cdot\mu_B(E_2)}{\mu_{basis\,2}(E_2)\cdot\mu_{basis\,1}(E_1) - \mu_{basis\,2}(E_1)\cdot\mu_{basis\,1}(E_2)},$$

and

$$\omega = \frac{\mu_{basis\,2}(E_2)\cdot\mu_{MOI}(E_1) - \mu_{basis\,2}(E_2)\cdot\mu_B(E_1) + \mu_{basis\,2}(E_1)\cdot\mu_B(E_2) - \mu_{basis\,2}(E_1)\cdot\mu_{MOI}(E_2)}{\mu_{basis\,2}(E_2)\cdot\mu_{basis\,1}(E_1) - \mu_{basis\,2}(E_1)\cdot\mu_{basis\,1}(E_2)}.$$

This can be derived by inserting Equation 3 into Equation S7 and solving for iodine mass.

By placing Equations S13 and S14 into Equation 6, we can examine the percent elemental error, which is demonstrated graphically in Figure 5. Therefore, it can be shown that the error on the mass of iodine in the voxel is:

$$Elemental\ error = \frac{a \cdot \rho_{iodine} - \frac{a-\tau}{\omega} \cdot \rho_{ICM} \cdot MF_{iodine}}{\frac{a-\tau}{\omega} \cdot \rho_{ICM} \cdot MF_{iodine}} \cdot 100\% \qquad (S15)$$

Clinical examples

Single-Energy Clinical Relevance

To understand this for clinical uses, it would be helpful to have a sort of “look-up” table for what has happened to a voxel given an observed change in CT number. Table A1 details examples that may be useful in clinical practice with a starting HAP value (m_1) of 0.126 g. These values are based on clinical levels of HAP (0-0.500 g/cm^3 of HAP) and an MOI of HAP, with tissue composition from [2] and mass attenuation coefficients from NIST XCOM [3].

Suppose we have a sample of soft tissue. We record $CT_{\#1} = 165\ HU$, then add more HAP and record $CT_{\#2} = 330$. Using the conventional methodology of determining HAP concentration, we would we assume that the HAP mass change is equivalent to the CT number change. A CT number ratio of $\frac{330\ HU}{165\ HU} = 2$, so we would incorrectly assume that we have a doubling of HAP mass in the voxel. Below we calculate the true HAP mass.

If we assume a starting mass of 0.126 g HAP (m_1), then we can calculate the actual mass change using Equations S1, S2, and S3:

$$X = \frac{CT_{\#2} \cdot \mu_{H_2O} - (\mu_B - \mu_{H_2O}) \cdot 1000}{CT_{\#1} \cdot \mu_{H_2O} - (\mu_B - \mu_{H_2O}) \cdot 1000} \qquad (S16)$$

Equation S16 can be substituted into Equation 8 to obtain the new mass.

$$m_2 = m_1 \left[\frac{CT_{\#2} \cdot \mu_{H_2O} - \left(\mu_B - \mu_{H_2O}\right) \cdot 1000}{CT_{\#1} \cdot \mu_{H_2O} - \left(\mu_B - \mu_{H_2O}\right) \cdot 1000}\right] \qquad (S17)$$

Using Equation S17, we can then calculate the true new mass.

$$m_2 = 0.126g \left[\frac{330\ HU \cdot 0.1929 \frac{1}{cm} - \left(0.2031 \frac{1}{cm} - 0.1929 \frac{1}{cm}\right) \cdot 1000\ HU}{165\ HU \cdot 0.1929 \frac{1}{cm} - \left(0.2031 \frac{1}{cm} - 0.1929 \frac{1}{cm}\right) \cdot 1000\ HU}\right] = 0.311g$$

From this, we can see the difference between the assumed new mass (0.252 g) and the true new mass (0.311 g). Using Equation S3, this then gives us an error of -19.1%, which is the same as can be seen in Table S1.

Of course, in practice, the initial HAP mass is generally not known, unless some kind of reference is included in the scan (e.g., in a HAP calibration phantom there will be several rods of known and varying HAP density with the same background material), and therefore the use of single-energy scanning can never be a perfect method for estimating HAP density, though the mass will be more accurate using Equation A17 than Equation 7. Similarly, other materials commonly quantified in the body like bone mineral density or iodine would show errors as shown here for HAP.

Spectral Decomposition Clinical Relevance

For spectral imaging, Table S2 allows a user to measure the “a” term (i.e., iodine basis vector length) in an iodine-water image and look up the corresponding iodine mass given a voxel of varying background material. Table S2 contains values based on tissue compositions and standard densities taken from reference [2] and mass attenuation coefficients from NIST XCOM [3], as well as the ICM of OMNIPAQUE™ 350 [1] and energy levels of 58 and 85 keV (corresponding to beams of 80 and 140 kV, respectively [4]).

Note that the bone material has very large errors because of its dissimilarity to water, the other clinically relevant tissues are much more similar to water, and therefore the error on the decomposition is smaller. Bone contains large portions of calcium, which is not water-like and therefore the error in an iodine-water decomposition is large.

For example, suppose we have a voxel of blood and OMNIPAQUE™ 350 [1], and we use energies of 80 and 140 kV to scan the voxel and an iodine-water basis to decompose the image. Suppose also that the voxel has a VF_{MOI} of 0.02 (i.e., a mass of 0.007 g of iodine in the voxel). We can use Equation S7 to determine the “a” term that we would obtain on the iodine-water image,

$$a = \frac{\varepsilon \cdot \mu_{water}(85\ keV) - \delta \cdot \mu_{water}(58keV)}{\mu_{water}(85keV) \cdot \mu_{iodine}(58keV) - \mu_{water}(58keV) \cdot \mu_{iodine}(85keV)}$$
$$= \frac{0.2799\frac{1}{cm} \cdot 0.1799\frac{1}{cm} - 0.2104\frac{1}{cm} \cdot 0.2092\frac{1}{cm}}{0.1799\frac{1}{cm} \cdot 40.904\frac{1}{cm} - 0.2092\frac{1}{cm} \cdot 14.72\frac{1}{cm}} = 0.0015.$$

If we incorrectly multiply “a” by the elemental density of iodine and the voxel size (1 cm^3) as in Equation S13, then we obtain,

$$m_{assumed} = 0.0015 \cdot 4.93\frac{g}{cm^3} \cdot 1\ cm^3 = 7.395\ mg\ iodine$$

However, if we use Equation S14 to calculate the real iodine mass, then we obtain the actual iodine mass in the voxel,

$$m_{real} = \frac{a - \tau}{\omega} \cdot \rho_{ICM} \cdot MF_{iodine} \cdot VS = \frac{0.0015 - 0.000075}{0.071} \cdot 1.406\frac{1}{cm^3} \cdot 0.2489 \cdot 1cm^3$$
$$= 7\ mg\ iodine.$$

If we compare the assumed mass (7.395 mg) with the actual mass (7 mg), this gives an error of 5.64% or 0.395 mg.

In practice, the exact composition of the background will not be known, and therefore the exact mass will not be possible to accurately calculate but based on the location of the voxel in the body, a user can make an educated guess as to its composition and use Equation S14 to estimate the elemental mass.

References

1. Federal Drug Administration. OMNIPAQUE^{TM} (iohexol) Injection. Reference ID: 4080358.
2. International Commission on Radiation Units and Measurements (ICRU). Tissue Substitutes in Radiation Dosimetry and Measurement, Report 44 of the International Commission on Radiation Units and Measurements. 1989.
3. Berger MJ, Hubbell JH, Seltzer SM, Chang J, Coursey JS, Sukumar R, Zucker DS, Olsen K. XCOM: Photon Cross Sections Database. NIST Standard Reference Database 8 (XGAM). 1998.
4. Jiang Hsieh. Computed Tomography: Principles, Design, Artifacts, and Recent Developments. Fourth Edition. Chapter 12: Dual-Energy and Spectral CT. SPIE Press, Bellingham, Washington, USA. 2022.

Table S1: Lookup table comparing errors in hydroxyapatite (HAP) mass assuming CT number is linear with HAP mass for differing amounts of HAP mass additions and background materials. The exact CT numbers, masses, and errors presented in this table are based on an MOI of HAP For other MOI, these values will differ. Assumed HAP mass change is based on the erroneous Equation 7, the actual HAP mass change uses Equation 8.

Tissue	Starting CT# [HU*]	Final CT# [HU*]	CT# Percent	Starting HAP** Mass [g]	Assumed Final HAP** Mass [g]	Actual Final HAP** Mass [g]	X Factor (mass multiplication factor)	Percent Error (between assumed and actual final HAP** mass)
**Vacuum**	-796	-398	50%	0.126	0.063	0.372	3.0	-83.1%
	-796	-1194	150%	0.126	0.189	-0.120	-0.95	-258%
	-796	-1592	200%	0.126	0.252	-0.366	-2.9	-169%
	-796	-1990	250%	0.126	0.315	-0.612	-4.9	-152%
	-796	-2388	300%	0.126	0.378	-0.857	-6.8	-144%
**Adipose**	36	18	50%	0.126	0.063	0.106	0.84	-40.5%
	36	54	150%	0.126	0.189	0.146	1.2	29/3%
	36	72	200%	0.126	0.252	0.166	1.3	41.6%
	36	90	250%	0.126	0.315	0.186	1.5	69.0%
	36	108	300%	0.126	0.378	0.207	1.6	83.1%
**Water**	113	56.5	50%	0.126	0.063	0.063	0.5	0%
	113	169.5	150%	0.126	0.189	0.189	1.5	0%
	113	226	200%	0.126	0.252	0.252	2.0	0%
	113	282.5	250%	0.126	0.315	0.315	2.5	0%
	113	339	300%	0.126	0.378	0.378	3.0	0%
**Muscle**	155	77.5	50%	0.126	0.063	0.039	0.31	62.4%
	155	232.5	150%	0.126	0.189	0.213	1.7	-11.4%
	155	310	200%	0.126	0.252	0.300	2.4	-16.1%
	155	387.5	250%	0.126	0.315	0.388	3.1	-18.7%
	155	465	300%	0.126	0.378	0.475	3.8	-20.4%
**Lung**	157	78.5	50%	0.126	0.063	0.038	0.30	67.5%
	157	235.5	150%	0.126	0.189	0.214	1.7	-11.8%
	157	314	200%	0.126	0.252	0.303	2.4	-16.8%
	157	392.5	250%	0.126	0.315	0.391	3.1	-19.5%
	157	471	300%	0.126	0.378	0.480	3.8	-21.2%
**Soft**	165	82.5	50%	0.126	0.063	0.033	0.26	89.3%
	165	247.5	150%	0.126	0.189	0.219	1.7	-13.6%
	165	330	200%	0.126	0.252	0.311	2.5	-19.1%
	165	412.5	250%	0.126	0.315	0.404	3.2	-22.1%

	165	495	300%	0.126	0.378	0.497	3.9	-23.9%
**Blood**	168	84	50%	0.126	0.063	0.032	0.25	97.2%
	168	252	150%	0.126	0.189	0.220	1.7	-14.1%
	168	336	200%	0.126	0.252	0.314	2.5	-19.8%
	168	420	250%	0.126	0.315	0.408	3.2	-22.8%
	168	504	300%	0.126	0.378	0.502	4.0	-24.7%

*HU = Hounsfield units

**HAP = hydroxyapatite

Table S2: Lookup table comparing errors in iodine mass erroneously assuming a decomposition method where the voxel is purely made of iodine and water. The exact "a" values, masses, and errors presented in this table are based on an ICM of OMNIPAQUE™ 350 [1]. For other ICM concentrations, these values will differ.

Tissue	"a" term	VF_{ICM}*	Assumed Iodine Mass [g]	Actual Iodine Mass [g]	Percent Error (between assumed and actual iodine mass)
**Vacuum**	0.0007	0.01	0.0035	0.0035	0.175%
	0.0014	0.02	0.007	0.007	0.175%
	0.0036	0.05	0.0175	0.0175	0.175%
	0.0057	0.08	0.028	0.028	0.175%
	0.0071	0.10	0.0351	0.035	0.175%
**Adipose**	0.0005	0.01	0.0023	0.0035	-33.1%
	0.0012	0.02	0.0059	0.007	-16.3%
	0.0033	0.05	0.0164	0.0175	-6.21%
	0.0055	0.08	0.027	0.028	-3.69%
	0.0069	0.10	0.034	0.035	-2.85%
**Water**	0.0007	0.01	0.0035	0.0035	0.175%
	0.0014	0.02	0.007	0.007	0.175%
	0.0036	0.05	0.0175	0.0175	0.175%
	0.0057	0.08	0.028	0.028	0.175%
	0.0071	0.10	0.0351	0.035	0.175%
**Muscle**	0.0007	0.01	0.0036	0.0035	4.15%
	0.0015	0.02	0.0071	0.007	2.14%
	0.0036	0.05	0.0176	0.0175	0.939%
	0.0057	0.08	0.0281	0.028	0.638%
	0.0071	0.10	0.0351	0.035	0.537%
**Lung**	0.0007	0.01	0.0036	0.0035	2.79%
	0.0014	0.02	0.0071	0.007	1.47%
	0.0036	0.05	0.0176	0.0175	0.677%
	0.0057	0.08	0.0281	0.028	0.479%
	0.0071	0.10	0.0351	0.035	0.413%
**Soft**	0.0007	0.01	0.0037	0.0035	4.78%
	0.0015	0.02	0.0072	0.007	2.46%
	0.0036	0.05	0.0177	0.0175	1.06%
	0.0057	0.08	0.0282	0.028	0.710%
	0.0071	0.10	0.0352	0.035	0.594%
**Blood**	0.0008	0.01	0.0038	0.0035	8.60%
	0.0015	0.02	0.0073	0.007	4.34%
	0.0036	0.05	0.0178	0.0175	1.79%
	0.0057	0.08	0.0283	0.028	1.15%
	0.0072	0.10	0.0353	0.035	0.941%
**Bone**	0.0073	0.01	0.036	0.0035	929%

	0.0079	0.02	0.0392	0.007	460%
	0.0099	0.05	0.0487	0.0175	178%
	0.0118	0.08	0.0582	0.028	108%
	0.0131	0.10	0.0646	0.035	84.6%

*VF_{ICM} = volume fraction of the iodinated contrast material (ICM)
